# Mapping Subchondral Bone Density Distribution in the Canine C6-C7 Vertebral Endplates: A CT-OAM Study

**DOI:** 10.3390/ani13223432

**Published:** 2023-11-07

**Authors:** Vincenz Kramer, Peter Böttcher

**Affiliations:** Small Animal Clinic, Freie Universität Berlin, 14163 Berlin, Germany; peter.boettcher@fu-berlin.de

**Keywords:** CTOAM, canine, cervical spine, bone density, subsidence, cervical spondylomyelopathy, cage

## Abstract

**Simple Summary:**

Treating cervical spinal cord compression in dogs can be complex, especially when dynamic instability is involved, a concern not fully addressed by standard surgery. A common approach includes using intervertebral cages to support and stabilize the affected area. However, the risk of cage subsidence into the vertebral endplates can undermine treatment effectiveness. In this study, we used computed tomography osteoabsorptiometry to map the subchondral bone density in the C6-C7 vertebral motion unit of dogs. This density serves as an indicator of endplate mechanical stability. Similar to humans, we found that bone density within the endplate is unevenly distributed. The central and centro-dorsal regions have low bone density, making them less stable. This correlates with the common site of cage subsidence. Our findings suggest a need to reconsider the design and placement of spinal cages. Positioning them on areas of higher bone density, such as the ventral, dorsal, and lateral extensions of the vertebral endplates, may reduce the risk of subsidence, improving treatment outcomes.

**Abstract:**

Intervertebral cage subsidence is a common complication in treating disc-associated cervical spondylomyelopathy in dogs. The mechanical stability of the vertebral endplate in contact with the cage is crucial to preventing subsidence. This study aims to assess subchondral bone mineral density (sBMD) in the canine vertebral endplate (specifically, the C6-C7 vertebral motion unit) as a measure of its mechanical stability. The sBMD distribution was mapped for the C6 caudal and C7 cranial vertebral endplates in 15 middle- to large-breed dogs using computed tomography osteoabsorptiometry. The sBMD distribution in the canine C6 and C7 vertebral endplates exhibited a heterogeneous pattern, with lower density observed in the central and dorsal contact areas of the nucleus pulposus, where common subsidence occurs. Our results suggest a potential need to redesign intervertebral cages to ensure that contact areas align with regions of higher bone density. A broad-based design extending toward the lateral and dorsal aspects of the annulus fibrosus contact area may enhance stability.

## 1. Introduction

Disc-associated cervical spondylomyelopathy (DA-CSM) is a prevalent and debilitating condition in dogs, characterized by the compression of the spinal cord and nerve roots in the cervical region [1,2]. Among the various locations where disc-associated cervical spinal cord compression occurs, the C5-C6 and C6-C7 levels are most frequently affected. A frequently employed therapeutic approach for DA-CSM often involves surgical intervention, which includes techniques for distraction, stabilization, and fusion [3,4,5,6,7]. A crucial component of this surgical strategy is the utilization of intervertebral cages as spacers. These cages play a pivotal role in maintaining the desired distraction until successful fusion between adjacent vertebrae is achieved, ensuring long-term stability of the treated cervical articulation.

However, one of the most frequently encountered complications in cervical fusion procedures is cage subsidence. Subsidence refers to the settling of an intervertebral cage into the adjacent vertebral endplate(s) [3,5,8,9,10,11,12]. This phenomenon can result in the reduction of intervertebral space, potentially leading to the compression of nearby neural structures, decreased segmental stability, and even the recurrence of clinical symptoms. Several factors contribute to the occurrence of cage subsidence, including inadequate sizing of the cage, improper placement, insufficient bone quality, and excessive load-bearing forces on the cage [13]. Given the critical implications of cage subsidence, assessing and mitigating this risk is paramount for achieving successful outcomes in cervical fusion procedures.

It is noteworthy that in human patients undergoing anterior cervical discectomy and fusion, which shares similarities with the canine surgical approach for cervical distraction, stabilization, and fusion, subsidence represents the most common hardware-related complication [12,14]. Its incidence in humans is reported to range from 0% to as high as 83%, with a mean rate of 21.1% [14]. In the context of dogs, studies have documented cage subsidence across a wide spectrum, with incidence rates spanning from 11% to 100% in treated spinal articulations [3,4,5,8,9,10,11,15,16,17,18,19]. Among these investigations, a consistent finding is that the cranial endplate of the caudal vertebra is the most commonly affected site.

Addressing cage subsidence necessitates considering both the design of the cage itself and its placement on the vertebral endplate(s). A cage design that facilitates widespread contact and even pressure distribution on the endplate can significantly reduce the risk of subsidence, irrespective of the forces acting on the functional unit. Furthermore, taking into account the inherent mechanical strength variations within different regions of the endplates and their alignment with the cage design is of paramount importance. The load/contact exerted on areas with lower bone density, which are more prone to subsidence, could have detrimental consequences. Conversely, a cage design that considers these topographic variations may offer potential strategies to mitigate the risk of subsidence.

Another critical consideration is the choice of cage material. Among the alternatives to steel or titanium, polyetheretherketone (PEEK) has gained popularity as an intervertebral spacer in human medicine since receiving medical-grade approval from the Food and Drug Administration in 1998. While PEEK offers advantages in terms of mechanical properties, it is inherently bioinert and lacks the ability to integrate with bone tissue [15]. Initially, there was limited clinical and radiographic evidence favoring PEEK cages over bone grafts and titanium cages in human cervical spine surgeries [16]. However, the application of surface coatings to these implants has shown significant improvements in fusion rates, as demonstrated in a canine spinal model [17]. Notably, in that study, which involved seven mature beagles with 14 PEEK cages, subsidence was not observed. This positive result could be linked to the tailored design of the implant, crafted to match the intervertebral space, the robust fixation achieved through the integration of screws within the implant, and the advantageous elastic modulus exhibited by PEEK.

Custom-designed cages have previously demonstrated success in dogs with DA-CSM [18,19]. The enhancement of these designs primarily revolved around molding the cage to closely match the curvature of the cranial and caudal endplates. While one design aimed to cover a larger portion of the endplate area to enhance further biomechanical compatibility, both designs encountered varying degrees of subsidence. Consequently, it becomes evident that accounting not only for the shape of the vertebral endplates but also for their strength and capacity to resist subsidence is crucial in the application of intervertebral cages.

Computed tomographic osteoabsorptiometry (CT-OAM), a technique for measuring the bone mineral density of the subchondral bone plate through maximum intensity projection, reflects the long-term loading history of the joint [20]. Typically, CT-OAM values are expressed in Hounsfield Units (HU). The distribution of bone mineral density in the vertebral endplates assessed using CT-OAM has been strongly correlated with the local mechanical strength of the endplates in humans, as determined by indentation testing [21,22,23,24,25]. Reversely, by mapping the subchondral bone density of the endplates, we gained direct insights into their mechanical strength and the potential implications for cage design. Studies conducted on the human cervical spine have revealed a significant variation in bone density within the vertebral endplates [25,26]. One of those studies also reported significant differences in the distribution of the subchondral bone mineral density (sBMD) between superior and inferior endplates [26], suggesting a need for a non-uniform cage design.

While there is extensive research available in human medicine, there is no published data concerning the sBMD and strength of canine cervical vertebral endplates. Therefore, this study’s primary objective is to map the sBMD of vertebral endplates within the C6-C7 vertebral motion unit (VMU) of the canine cervical spine using CT-OAM. The findings obtained from this mapping have the potential to provide valuable insights into the stability of those endplates and could contribute to the enhancement of current cage designs used in dogs affected by DA-CSM at the C6-C7 level.

## 2. Materials and Methods

### 2.1. Specimen Preparations

Fifteen canine cadaveric C6-C7 VMUs from adult dogs weighing between 20 and 45 kg were investigated. The dogs were client-owned and euthanized for reasons unrelated to the study, with no known effect on bone density. For final inclusion, the VMU had to be free of any pre-existing, CT-detected, or gross anatomical pathology. When reviewing the axial CT images for any abnormalities, we also considered focal vertebral changes that have been recently described in healthy dogs as exclusion criteria [27].

**Figure 1:**
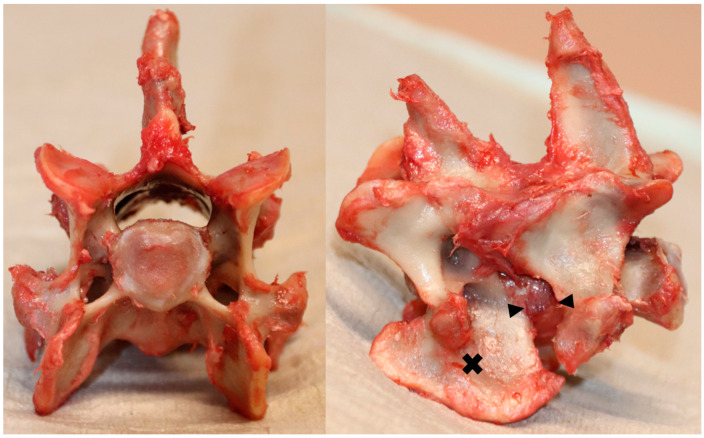
An illustrative instance of dissected C6-C7 vertebral motion unit (VMU); (**left**) Axial view highlighting the cranial aspect of the C6 vertebral endplate; (**right**) Lateral view indicating C6 with a cross marker and outlining the intervertebral disc with arrows.

After euthanasia, the cervical spine, starting from the second cervical vertebra to the second thoracic vertebra, was harvested, placed in double-sealed plastic bags, and stored at −20 °C. On the day of the final investigation, the spine was thawed to room temperature, and the C6-C7 VMU was isolated by disarticulation at C5-C6 and C7-Th1. All surrounding soft tissues were removed, except for the intervertebral disc, ventral and dorsal longitudinal ligaments, ligamentum flavum, and joint capsules of the facet joints (see Figure 1).

### 2.2. CT-OAM Mapping

The VMU C6-C7 was positioned in a neutral position on a foam block, and axial CT imaging was performed (Philips Brilliance; Philips Medical Systems, Best, The Netherlands) with a slice thickness of 0.8 mm and an increment of 0.4 mm. The CT data was loaded into ParaView (Paraview 5.11.0, Kitware Inc., Clifton Park, NY, USA), and a 3D surface model of the C6-C7 VMU was generated. Within 3-Matic (Materialise NV, Leuven, Belgium), the C6 and C7 vertebrae were isolated and saved as separate surface models.

For establishing topographic correspondence between all 15 caudal C6 and cranial C7 endplates, visual inspection was conducted on all C6-C7 endplate pairs, and the VMU that best resembled the mean anatomical shape was chosen as a reference for subsequent image registration. On the CT data of the reference VMU, the caudal endplate of C6 and the cranial endplate of C7 were manually segmented and used as targets for non-rigid volume image matching of the other 14 CT data sets using 3D-slicer [28] and the Elastix module [29,30].

Following volume image registration, the subchondral bone density of the caudal C6 and cranial C7 endplates of each of the 15 vertebrae were analyzed using maximum intensity projection (MIP) through a Python script within ParaView. The extracted CT-OAM values were then projected onto the 3D surface models of the caudal C6 and cranial C7 reference endplates. This assured topographic correspondence between the CT-OAM analysis of each individual C6-C7 VMU. In the end, we obtained 15 models of the C6 and C7 reference end plates, colored with the CT-OAM values of the 15 VMU. For further statistical analysis, the individual CT-OAM values were exported, with each data point corresponding to the same anatomical point on the vertebral endplate across all 15 VMUs.

**Figure 2:**
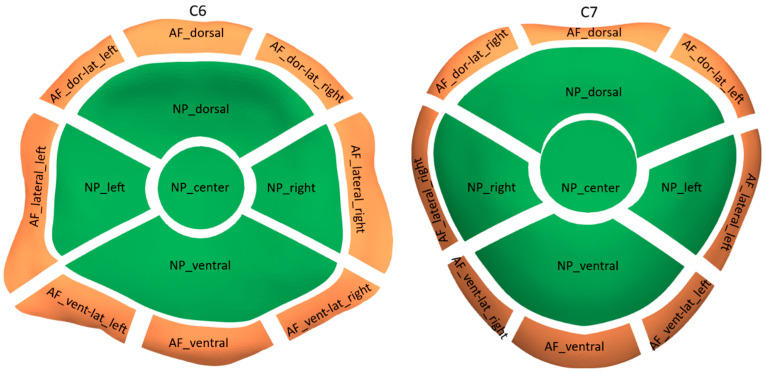
Partitions of vertebral endplates (AS = annulus fibrosus, NP = nucleus pulposus).

To enable a topographic description of the distribution of the obtained CT-OAM values within the vertebral endplates, the reference VMU was subjected to axial MRI scanning (T2 weighted sequence). Following manual segmentation of the annulus fibrosus (AF) and the nucleus pulposus (NP), the C6 and C7 reference endplate models were partitioned accordingly (see Figure 2). Subsequently, each partition was further divided into anatomically functional subdivisions, resulting in a total of eight subdivisions for the AF contact area and five subdivisions for the NP contact area for each of the two endplate models.

### 2.3. Statistical Analysis

To assess the normality of the CT-OAM values, the Kolmogorov-Smirnov test was employed, revealing a normal distribution. Consequently, the CT-OAM values are presented as the mean along with the corresponding standard deviation (SD) or minimal and maximal values, if deemed appropriate. Variations in topography were examined using a paired sample *t*-test. For comparisons between the C6 and C7 vertebral endplates, as well as AF and NP partitions, a significance level of ≤0.05 was adopted. Moreover, to account for multiple tests, a Bonferroni correction was applied, and a significance level of ≤0.01 was used for all subsequent statistical comparisons involving subdivisions and their aggregate groups.

## 3. Results

A total of 15 VMUs were examined, which were collected from 8 female dogs (7 intact, 1 neutered) and 7 male dogs (all castrated), with an average age of 10.9 years (ranging from 6.5 to 16 years) and an average body weight of 34.1 kg (ranging from 22.0 to 55.0 kg). The sample comprised six mixed-breed dogs: two Retrievers, two Swiss Mountain Dogs, an Australian Shepherd, a Bordeaux Mastiff, a Boxer, a German Shepherd Dog, and a Doberman Pinscher.

**Figure 3:**
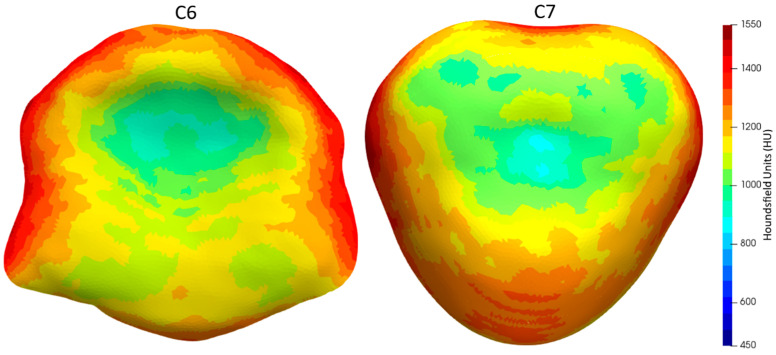
Color mapping of mean sBMD distribution of the caudal endplate of C6 (**left**) and the cranial endplate of C7 (**right**) (*n* = 15).

**Figure 4:**
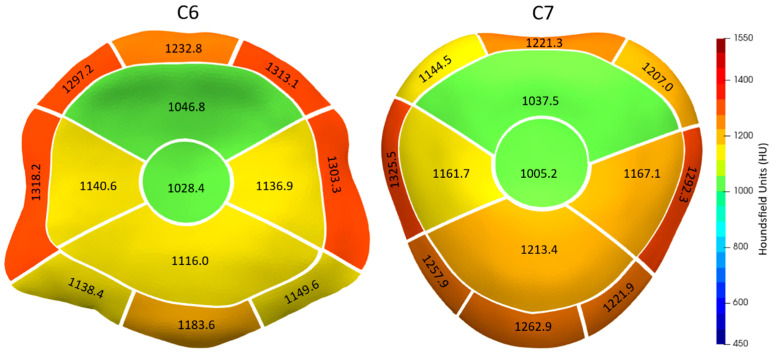
The mean sBMD for the topographic subdivisions of the caudal endplate of C6 (**left**) and the cranial endplate of C7 (**right**).

The sBMD of the caudal endplate of C6 and the cranial endplate of C7 displayed a distinct heterogeneous pattern. This was characterized by lower sBMD values at the center and higher values at the periphery (Figure 3 and Figure 4). The highest CT-OAM values for the C6 endplate were found in the lateral and dorsal regions of the AF, while the C7 endplate exhibited the opposite pattern, with maxima in the lateral and ventral AF regions. In both endplates, the region with the lowest sBMD was the center of the NP area, closely followed by the dorsal NP region.

Overall, there was no significant difference (*p* = 0.8408) in mean sBMD between the C6 (1169.9 HU, SD: 77.6) and C7 (1172.3 HU, SD: 72.2) endplates (see Table 1 and Table 2).

The AF area showed significantly higher sBMD values (1248.5 HU, SD: 81.5 for C6; 1245.5 HU, SD: 65.3 for C7) than the NP area (1092.6 HU, SD: 81.5 for C6; 1121.0 HU, SD: 86.8 for C7) for both C6 and C7 endplates (*p* < 0.0001 for both). However, there was no statistically significant difference in the sBMD of the AF and NP contact areas between C6 and C7 (*p* = 0.8120 for AF; *p* = 0.0662 for NP).

**Figure 5:**
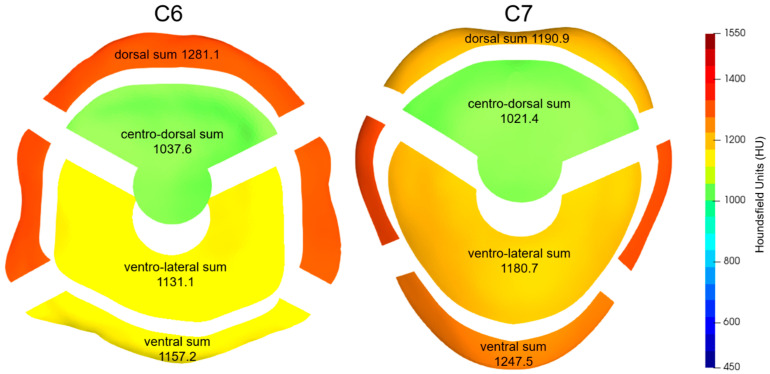
Mean sBMD for the aggregate groups of the subdivisions from Figure 4 with the caudal endplate of C6 (**left**) and the cranial endplate of C7 (**right**).

When the topographic subdivisions were aggregated into larger functional units (Figure 5), the dorsal region of the C6 AF had an average CT-OAM value of 1281.1 HU (SD: 73.9), which was significantly higher than the corresponding area of C7, which had a value of 1190.9 HU (SD: 60.7) (*p* < 0.0001). Conversely, the ventral region of the C6 AF had a mean CT-OAM value of 1157.2 HU (SD: 99.9), which was significantly lower than that of C7, which had a value of 1247.5 HU (SD: 84.2).

For the NP functional units, the centro-dorsal NP region of both C6 (1037.6 HU, SD: 89.9) and C7 (1021.4 HU, SD: 93.8) had significantly lower CT-OAM values compared to the ventro-lateral NP regions (1131.1 HU, SD: 85.3 for C6; 1180.7 HU, SD: 85.8 for C7) (*p* < 0.0001).

## 4. Discussion

Our findings reveal a complex and heterogeneous density pattern that mirrors certain aspects of the human cervical spine. Specifically, within the caudal C6 and cranial C7 canine endplates, we observe the highest bone density in the peripheral regions of the endplates. In contrast, the region corresponding to the NP and, more prominently, the central and dorso-central areas exhibit significantly lower bone density levels.

These results correlate with the occurrence of cage subsidence in the canine cervical spine reported in the literature, with subsidence often occurring at the central and dorso-central aspects of the NP [3,10,31,32].

The low subchondral bone density in the central and centro-dorsal aspects of the canine cervical vertebral endplates is comparable to the findings previously described for the human cervical spine [25,26]. However, there is a notable difference in the location of maximal sBMD between humans and canines. Dogs lack the maximum at the ventral aspect of the cranial endplate, a feature observed in humans [26]. This discrepancy may be attributed to differences in posture and neck biomechanics between bipedal (humans) and quadrupedal (canines) species. Since areas with high subchondral bone density are potential contact surfaces for spinal cage placement, caution should be exercised when interpolating cage design and surgical techniques from humans to canines.

Numerous studies conducted on human vertebrae have demonstrated significant positive correlations between CT-OAM values and the mechanical strength of the vertebral endplates when subjected to penetrating forces [23,24,25]. Therefore, non-destructive measurement of subchondral bone density provides a realistic estimate of the endplate’s strength. Recent human studies have also shown that higher sBMD in the endplates is associated with a lower likelihood of subsidence compared to endplates with lower sBMD and vice versa [21,33]. This further validates the use of CT-OAM as a valuable tool for predicting cage subsidence and optimization of cage design based on CT-OAM bone density mapping like in human spinal surgery [34].

The current techniques utilized in dogs for distraction, stabilization, and fusion primarily focus on preserving the lateral and dorsal portions of the AF, with cage or spacer devices placed in the NP area and the ventral region of the AF. This approach has multiple factors influencing its adoption: preserving a significant portion of the AF is intended to maintain intervertebral stability as much as possible. Preservation of the lateral arc of the AF helps prevent nerve damage and bleeding from the vertebral artery. Additionally, sparing the dorsal part of the AF prevents unintended entry into the vertebral canal, which could potentially lead to spinal cord damage and bleeding from the venous plexus. While these considerations have become widely accepted, the design of current cervical spinal cages in dogs and their recommended placement within the intervertebral space conflict with the distribution of bone density described hereby and the presumably related strength of the endplates in contact with the cage. Based on the presented finding of sBMD distribution, to prevent cage subsidence, cervical spinal cages should ideally rest on both the ventral and dorsal aspects of the vertebral endplates and should extend as far laterally as possible. On the contrary, the central and centro-dorsal aspects, which are recognized as the least stable regions of the cervical endplates, should be avoided and not be regarded as significant load-bearing surfaces for spinal cages. The concern regarding an increased risk of vertebral instability associated with aggressive (dorsal and potentially lateral) resection of the AF, is likely mitigated by the established combination of intervertebral cage placement and fixation with locking screws or ventral plating because such techniques provide significant stability to the fused VMU [35,36]. Furthermore, the potential complications of penetrating the vertebral canal when sacrificing the dorsal AF may still be limited by the presence of the dorsal longitudinal ligament. Additionally, the dorsal longitudinal ligament plays a pivotal role in stabilizing the VMU, further supporting the benefit of preserving this ligament [37].

We acknowledge several limitations in the current study. The findings and conclusions are specific to the C6-C7 VMU in elderly middle- to large-breed dogs. Studies conducted on humans have demonstrated substantial variations in sBMD along the cervical spine [26,34], which suggests the existence of analogous variations in dogs. Additionally, it is important to consider potential variations in small dogs and chondrodystrophic breeds [38]. Therefore, we do not advise generalizing the observed sBMD patterns to other VMUs or specific populations other than those investigated.

While castration does not appear to significantly affect bone metabolism in female dogs, it is worth noting that androgen deficiency may impact bone metabolism in male dogs [39]. It is possible that the results of our study were influenced by the reproductive status of the subjects, potentially making the investigated population less representative of the typical DA-CSM patient population. Nevertheless, the conclusions drawn from this study primarily rely on identifying areas of low and high bone density rather than assessing absolute values. Consequently, any systemic changes in bone density would probably have limited significance.

It is crucial to acknowledge that sBMD patterns may vary in VMUs affected by DA-CSM, often due to the presence of vertebral endplate sclerosis. Therefore, it is essential to compare the presented results with data from affected VMUs to ensure their practical applicability. Furthermore, incorporating biomechanical testing and, ultimately, clinical evaluation of the optimized cage design is vital. This comprehensive approach ensures that any new cage design takes into account not only bone density distribution, including topographic and breed-related variations but also potential mechanical differences between various breeds and VMU levels [38].

Because osteoporosis, comparable to humans, is not a common finding in elderly dogs, we are confident that the described findings hold true for mature dogs overall. Further research is necessary to investigate these aspects and gain a more comprehensive understanding.

Lastly, it should be noted that the speculations regarding the mechanical strength of the investigated vertebral endplates are based on the assumption that there exists a similar correlation between CT-OAM values and endplate strength in dogs as reported in humans [21,26,34]. However, to gain a more accurate understanding, dedicated indentation testing on canine vertebral endplates will be necessary. Conducting validation studies of this kind would additionally offer insights into the clinical implications of the observed variations in sBMD within the canine C6 and C7 vertebral endplates, particularly concerning their potential impact on resistance against cage subsidence. Drawing insights from a human vertebrae study [25], a variability in sBMD ranging from 50 to 100 HU equates to a significant discrepancy in the force necessary to breach the vertebral endplate. In dogs, considering the mean CT-OAM values of 1021.4 HU and 1037.6 HU for the centro-dorsal NP partitions of the C6 and C7 endplates, respectively, compared to the adjacent ventral NP area and the entire AF contact area, there exists a difference of over 140 HU. This substantial divergence strongly suggests a notable variance in bone stiffness and resistance to penetrating forces and therefore a high risk of cage subsidence at these sites. In contrast, the insignificant difference in sBMD of only 16.2 HU between the C7 and the C6 centro-dorsal NP aggregation does not explain the observation that cage subsidence affects predominately the cranial endplate of the caudal vertebra [3,5,10,11,32]. Other factors, such as significant differences in contact pressure acting on the cranial and caudal endplates have to be assumed.

Considering all the limitations mentioned, this study can only represent an initial step toward optimizing cage design. Future research endeavors will need to confirm the significance of CT-OAM values in assessing the resistance of canine vertebral endplates to subsidence, encompassing destructive indentation testing. Once the postulated strong correlation has been confirmed, it will be necessary to validate the described CT-OAM patterns in DA-CSM-affected VMUs to ensure their applicability in clinical cases. Furthermore, CT-OAM mapping should be extended to other clinically relevant cervical spine levels, and subsequent studies should encompass chondrodystrophic and small-breed dogs to enable the broad application of CT-OAM mapping in the optimization of cage designs for canine spine surgery.

Ultimately, achieving a maximal reduction in the occurrence of cage subsidence will likely necessitate a multifaceted approach that considers various factors, including cage design, material properties, and methods of fixation.

## 5. Conclusions

The distribution of subchondral bone density in the caudal C6 and cranial C7 vertebral endplates reveals a distinct heterogeneity, with the lowest density consistently observed around the customary sites of cage subsidence—most notably, the central and centro-dorsal regions of the nucleus pulposus contact area. Currently, high-density regions within the endplates remain underutilized as load-bearing zones for cervical cages or spacers in canine patients. Future cage designs could incorporate a broader coverage of the lateral and dorsal peripheries of the endplates while deliberately avoiding the central and dorsal NP regions. To ensure the applicability of the presented findings in clinical settings, it is imperative to conduct additional research and validation. This includes biomechanical testing and the inclusion of study populations affected by DA-CSM.

## Figures and Tables

**Table 1:** CT-OAM values with their corresponding SD for C6, its partitions, subdivisions, as well as their aggregate groups.

C6									
1169.9									
77.6	AF								
	1248.5								
	81.5	AF-Dorsal-Sum			AF-Vental-Sum			AF-Lateral-Left	AF-Lateral-Right
		1281.1			1157.2			1318.2	1303.3
		73.9			99.9			89.8	88.2
		AF-Dorsal-Left	AF-Dorsal-Center	AF-Dorsal-Right	AF-Ventral-Left	AF-Ventral-Center	AF-Ventral-Right		
		1297.2	1232.8	1313.1	1138.4	1183.6	1149.6		
		78.2	79.5	81.8	105.1	104.8	111.4		
	NP								
	1092.6								
	81.5	NP-Centrodorsal-Sum		NP-Ventrolateral-Sum					
		1037.6		1131.1					
		89.9		85.3					
		NP-Dorsal	NP-Central	NP-Left	NP-Ventral	NP-Right			
		1046.8	1028.4	1140.6	1116.0	1136.9			
		76.1	109.3	92.6	85.3	84.9			

**Table 2:** CT-OAM values with their corresponding SD for C7, its partitions, subdivisions, as well as their aggregate groups.

C7									
1172.3									
72.2	AF								
	1245.5								
	65.3	AF-Dorsal-Sum			AF-Vental-Sum			AF-Lateral-Left	AF-Lateral-Right
		1190.9			1247.5			1292.3	1325.5
		60.7			84.2			75.4	73.8
		AF-Dorsal-Left	AF-Dorsal-Center	AF-Dorsal-Right	AF-Ventral-Left	AF-Ventral-Center	AF-Ventral-Right		
		1207.0	1221.3	1144.6	1221.9	1262.9	1257.9		
		65.1	75.6	67.6	86.6	98.4	86.8		
	NP								
	1121.0								
	86.8	NP-Centrodorsal-Sum		NP-Ventrolateral-Sum					
		1021.4		1180.7					
		93.8		85.8					
		NP-Dorsal	NP-Central	NP-Left	NP-Ventral	NP-Right			
		1037.5	1005.2	1167.1	1213.4	1161.7			
		95.3	96.7	87.4	93.4	84.4			

## Data Availability

The full data set can be made available upon request by the corresponding author.
